# Supplementary material for: Estimation of the force of infection and infectious period of skin sores in remote Australian communities using interval-censored data
Source: PLoS Comput Biol. 2020 Oct 5;16(10):e1007838. doi: 10.1371/journal.pcbi.1007838 (PMC7561265; doi:10.1371/journal.pcbi.1007838)
Supplement: S1 Text — MCMC diagnostics relating to convergence of the posterior distributions. (PDF) [file pcbi.1007838.s001.pdf]

MCMC Diagnostics

Here information relating to the diagnostics of the MCMC procedure is provided. Fig 1(A) shows that the posterior distributions from the PHN data have converged far from the prior distributions (which were $\mathcal{N} \sim (0.5, 0.5)$), and Fig 1(B) shows that the chains are well mixed. The same conclusion can be drawn from Fig 2 for the HH dataset and for the RC dataset in Fig 3.

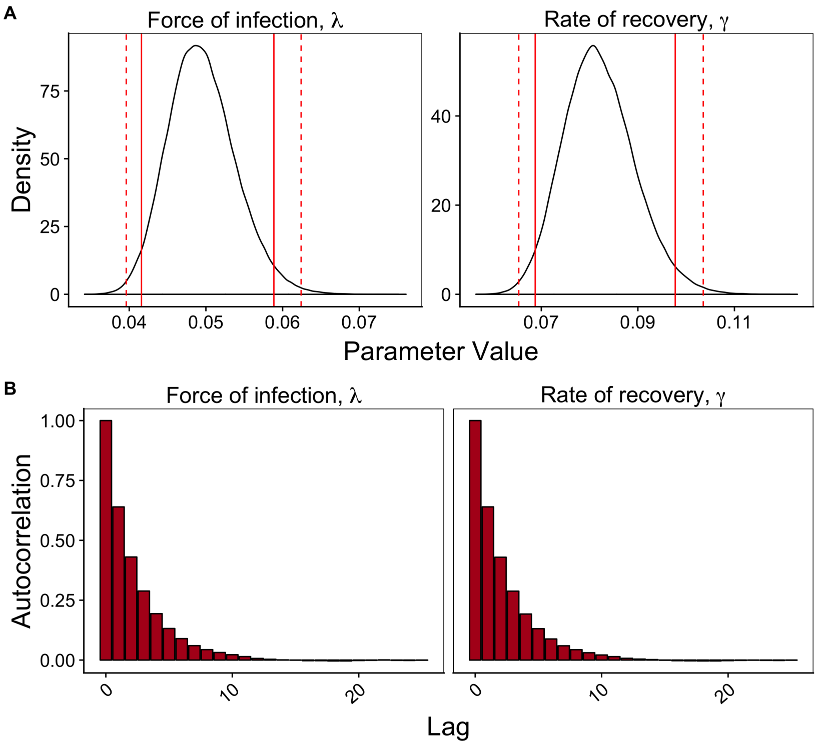

Fig 1. MCMC diagnostics for the PHN dataset. (A): Posterior density estimates of the force of infection, λ, and the rate of recovery, γ. The solid red line is a 95% credible interval, the dashed line a 99% credible interval. (B): Autocorrelation plots of the parameter values, for each of the chains.

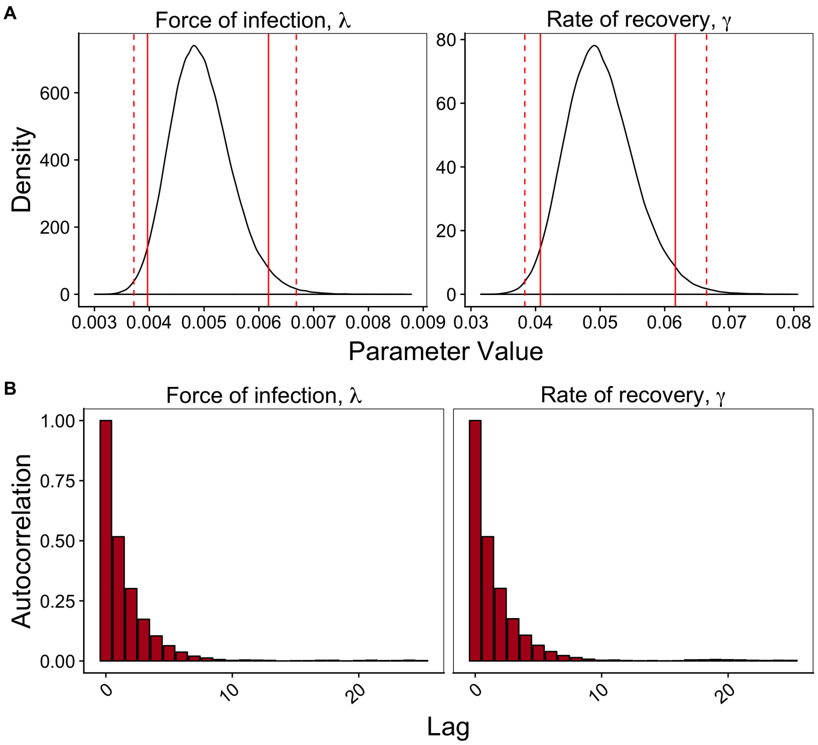

Fig 2. MCMC diagnostics for the HH dataset. (A): Posterior density estimates of the force of infection, λ, and the rate of recovery, γ. The solid red line is a 95% credible interval, the dashed line a 99% credible interval. (B): Autocorrelation plots of the parameter values, for each of the chains.

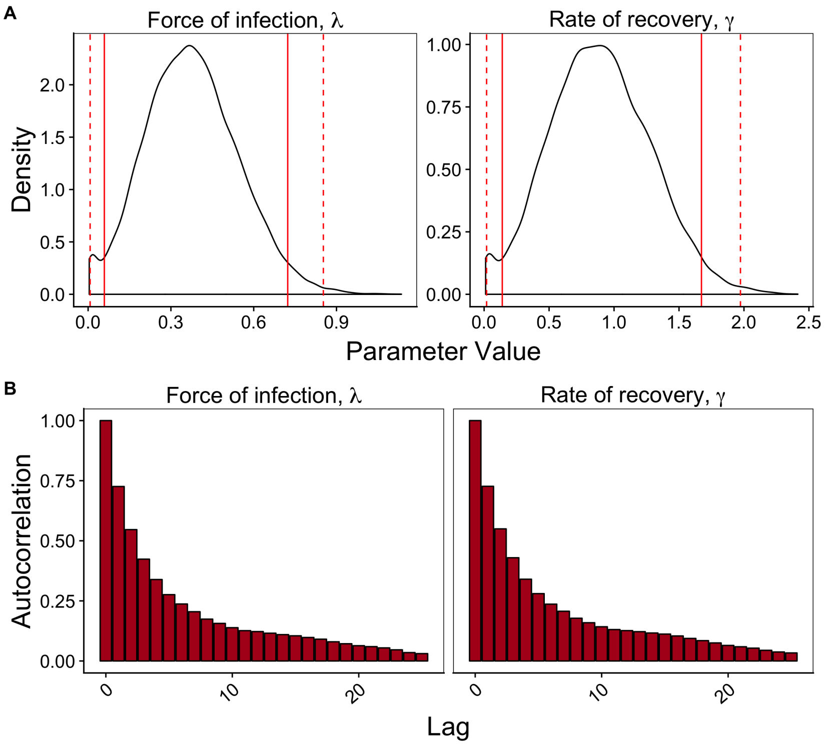

Fig 3. MCMC diagnostics for the RC dataset. (A): Posterior density estimates of the force of infection, λ, and the rate of recovery, γ. The solid red line is a 95% credible interval, the dashed line a 99% credible interval. (B): Autocorrelation plots of the parameter values, for each of the chains.
