# Supplementary material for: Estimation of the force of infection and infectious period of skin sores in remote Australian communities using interval-censored data
Source: PLoS Comput Biol. 2020 Oct 5;16(10):e1007838. doi: 10.1371/journal.pcbi.1007838 (PMC7561265; doi:10.1371/journal.pcbi.1007838)
Supplement: S2 Text — Derivation of the Fisher Information matrix for both variable and constant time between observations. (PDF) [file pcbi.1007838.s002.pdf]

Derivation of the Fisher Information matrix

The Fisher Information matrix is a representation of the amount of information that is contained in a model with parameters $\boldsymbol{\theta}$, about some observable value. The Fisher Information matrix is defined as,

$$I_{ij} = \mathbb{E}\left[\frac{\partial l(\boldsymbol{\theta})}{\partial \theta_i}\frac{\partial l(\boldsymbol{\theta})}{\partial \theta_j}\right].$$

Under some regularity conditions (which are assumed to be true), this is equivalent to,

$$I_{ij} = -\mathbb{E}\left[\frac{\partial^2 l(\boldsymbol{\theta})}{\partial \theta_i \theta_j}\right].$$

For the linearised SIS model, the Fisher Information matrix can be analytically determined and evaluated rapidly for a wide range of values for the time between each observation, $\boldsymbol{\delta}$. Here, only the case of a single individual is considered, but note that extension to N individuals simply results in the Fisher Information being multiplied by N, as there is an assumption that all individuals are identical.

Define the function,

$$\phi_S(\delta_i, \boldsymbol{\theta}) = \frac{\lambda - \lambda e^{-\delta(\gamma+\lambda)}}{\gamma + \lambda},$$

to be the probability that an individual is infected at time δ_i, given they were susceptible at time 0. Similarly, define,

$$\phi_I(\delta_i, \boldsymbol{\theta}) = \frac{\lambda + \gamma e^{-\delta(\gamma+\lambda)}}{\gamma + \lambda},$$

to be the probability that an individual is infected at time δ_i, given they were infected at time 0. For convenience, supress the dependence on $\boldsymbol{\theta}$ while deriving the Fisher Information matrix.

The likelihood function in Eq (4) can then be expressed as

$$L(\delta_i, \boldsymbol{\theta}) = \prod_i \Bigg[\Big((1 - \phi_S(\delta_i))^{\{X_i == S\}} \cdot \phi_S(\delta_i)^{\{X_i == I\}}\Big)^{\{X_{i-1} == S\}} \times \Big((1 - \phi_I(\delta_i))^{\{X_i == S\}} \cdot \phi_I(\delta_i)^{\{X_i == I\}}\Big)^{\{X_{i-1} == I\}}\Bigg], \quad (1)$$

where $\{X_i == S\}$ represents an indicator function. Taking the log of Eq (1) gives

$$l(\boldsymbol{\theta}) = \sum_i [\{X_{i-1} == S\} (\{X_i == S\} \log(1 - \phi_S(\delta_i)) + \{X_i == I\} \log(\phi_S(\delta_i)))$$
$$+ \{X_{i-1} == I\} (\{X_i == S\} \log(1 - \phi_I(\delta_i)) + \{X_i == I\} \log(\phi_I(\delta_i)))] . \quad (2)$$

The only terms of Eq (2) that contain $\boldsymbol{\theta}$ are the functions $\phi_S(\delta_i)$ and $\phi_I(\delta_i)$, and the log likelihood is linear in these functions. As such, the second partial derivatives of Eq (2) are simply

$$\frac{\partial^2 l(\boldsymbol{\theta})}{\partial \theta_i \theta_j} =$$
$$\sum_i \left[\{X_{i-1} == S\} \left(\{X_i == S\} \frac{\partial^2 \log(1 - \phi_S(\delta_i))}{\partial \theta_i \theta_j} + \{X_i == I\} \frac{\partial^2 \log(\phi_S(\delta_i))}{\partial \theta_i \theta_j} \right) \right.$$
$$\left. + \{X_{i-1} == I\} \left(\{X_i == S\} \frac{\partial^2 \log(1 - \phi_I(\delta_i))}{\partial \theta_i \theta_j} + \{X_i == I\} \frac{\partial^2 \log(\phi_I(\delta_i))}{\partial \theta_i \theta_j} \right) \right] . \quad (3)$$

The next step to determine the Fisher Information matrix is to consider the expectation of the product of the two random variables. In this case, X_i is Bernoulli, with probability of success (infection) of either $\phi_S(\delta_i)$ if $X_{i-1} = S$ or $\phi_I(\delta_i)$ if $X_{i-1} = I$. Using the law of total probability, it follows that

$$P(X_i = S \cap X_{i-1} = S) = P(X_i = S | X_{i-1} = S) P(X_{i-1} = S). \quad (4)$$

The first term of Eq (4) is simply the probability of failure (that is, not infected), given an individual was previously susceptible, which is $(1 - \phi_S)$. To calculate the second term, recall that the time between the ith and the $(i+1)$th observation is δ_i. Consider a discrete time Markov chain, with probability matrix

$$P_i = \begin{bmatrix} 1 - \phi_S(\delta_i) & \phi_S(\delta_i) \\ 1 - \phi_I(\delta_i) & \phi_I(\delta_i) \end{bmatrix} . \quad (5)$$

Then,

$$[P(X_r = S), P(X_r = I)] = [(1 - i_0, i_0)] \cdot \prod_{k=1}^{r} P_k$$

represents the probability that an individual is susceptible or infected at observation r, where i_0 represents the probability that an individual is initially infected, given here by the prevalence, I^*. For convenience, define $p_{s,r}$ to be the first element of this vector, and $p_{i,r} = 1 - p_{s,r}$ to be the second element.

As each X_i is Bernoulli, it follows from Eq (4) that the joint expectation is

$$E[\{X_i == S\}, \{X_{i-1} == S\}] = P(X_i = S \cap X_{i-1} = S) = (1 - \phi_S(\delta_i)) p_{s,i}, \quad (6)$$

and the other forms of this expectation follow similarly.

Finally, taking the expectation of Eq (3) gives

$$I_{\theta_1,\theta_2} = \sum_j p_{s,(j-1)} \cdot (1-\phi_S(\delta_j)) \frac{\partial^2 \log(1-\phi_S(\delta_j))}{\partial\theta_1\theta_2} + p_{s,(j-1)} \cdot \phi_S(\delta_j) \frac{\partial^2 \log(\phi_S(\delta_j))}{\partial\theta_1\theta_2} + p_{i,(j-1)} \cdot (1-\phi_I(\delta_j)) \frac{\partial^2 \log(1-\phi_I(\delta_j))}{\partial\theta_1\theta_2} + p_{i,(j-1)} \cdot \phi_I(\delta_j) \frac{\partial^2 \log(\phi_I(\delta_j))}{\partial\theta_1\theta_2}. \tag{7}$$

The expressions for each second derivative were found using Sage, and are implemented for numeric evaluation as part of the `TMI` package (`https://github.com/MikeLydeamore/TMI`).

0.1 Constant time between observations

If it is assumed that the time between observations is constant, that is,

$$t_{i+1} - t_i = \delta \ \forall i,$$

then the P matrix in Eq (5) is independent of i. Because of this, an analytic expression for P^r is [?],

$$P^r = \frac{1}{\phi_S + (1-\phi_I)} \begin{bmatrix} 1-\phi_I & \phi_S \\ 1-\phi_I & \phi_S \end{bmatrix} + \frac{(\phi_I - \phi_S)^r}{\phi_S + (1-\phi_I)} \begin{bmatrix} \phi_S & -\phi_S \\ \phi_I - 1 & \phi_I \end{bmatrix}.$$

The expression for the Fisher Information in Eq (7) is verified in the constant time between observations case again through a simulation estimation approach. For a given set of parameters, $\boldsymbol{\theta} = \{\lambda, \gamma\}$, 64 populations of the SIS model are simulated, and parameters estimated under each sample spacing, $\delta \in \{5, 10, \ldots, 40\}$. The determinant of the covariance matrix under each sample spacing is calculated and normalised. The results of this verification are shown in Fig 1.

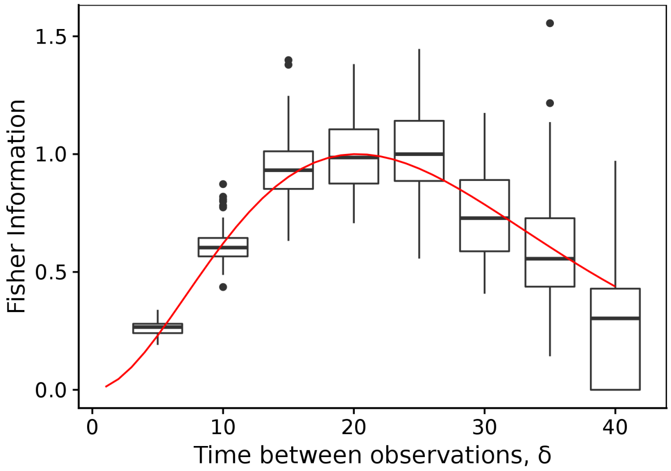

Fig 1. Numeric verification of the expressions for the Fisher Information matrix. The boxplot represents the summary of the determinants of the covariance matrix, and the red line the analytic expression for the Fisher Information matrix, with each curve normalised separately. The chosen parameters were $\lambda = 1/60$ and $\gamma = 1/20$.
