# Supplementary material for: Estimation of the force of infection and infectious period of skin sores in remote Australian communities using interval-censored data
Source: PLoS Comput Biol. 2020 Oct 5;16(10):e1007838. doi: 10.1371/journal.pcbi.1007838 (PMC7561265; doi:10.1371/journal.pcbi.1007838)
Supplement: S3 Text — Diagnostics of the optimisation of the sampling strategies. (PDF) [file pcbi.1007838.s003.pdf]

Optimal sampling strategy diagnostics

We utilise the induced natural selection heuristic for finding optimal sampling strategies [1]. We choose the following parameters:

- Initial designs, $D \sim U[1, 40]$, with $|D| = 2000$,
- Number of generations, $W = 50$,
- Pertubation function, $f(d|d') = \mathcal{TN}(d', 1)$,
- Acceptance criteria: retain top 40, 30, 20, 10 and 5 designs (10 times each),
- Newly sampled designs, m: 5, 10, 20, 30 and 40 designs (10 times each).

Presented here are diagnostics which give evidence of the convergence of the induced natural selection heuristic. Fig 1, 2 and 3 show that the Fisher information matrix has converged (panel A), and that the chosen values for the sampling intervals also appear to have converged (panel B) for the variable interval strategies. The same conclusion is drawn for the fixed interval strategies, as shown in Figurse 4, 5 and 6.

References

1. Price DJ, Bean NG, Ross JV, Tuke J. An Induced Natural Selection Heuristic for Finding Optimal Bayesian Experimental Designs. Computational Statistics & Data Analysis. 2018;126:112–124. doi:10.1016/j.csda.2018.04.011.

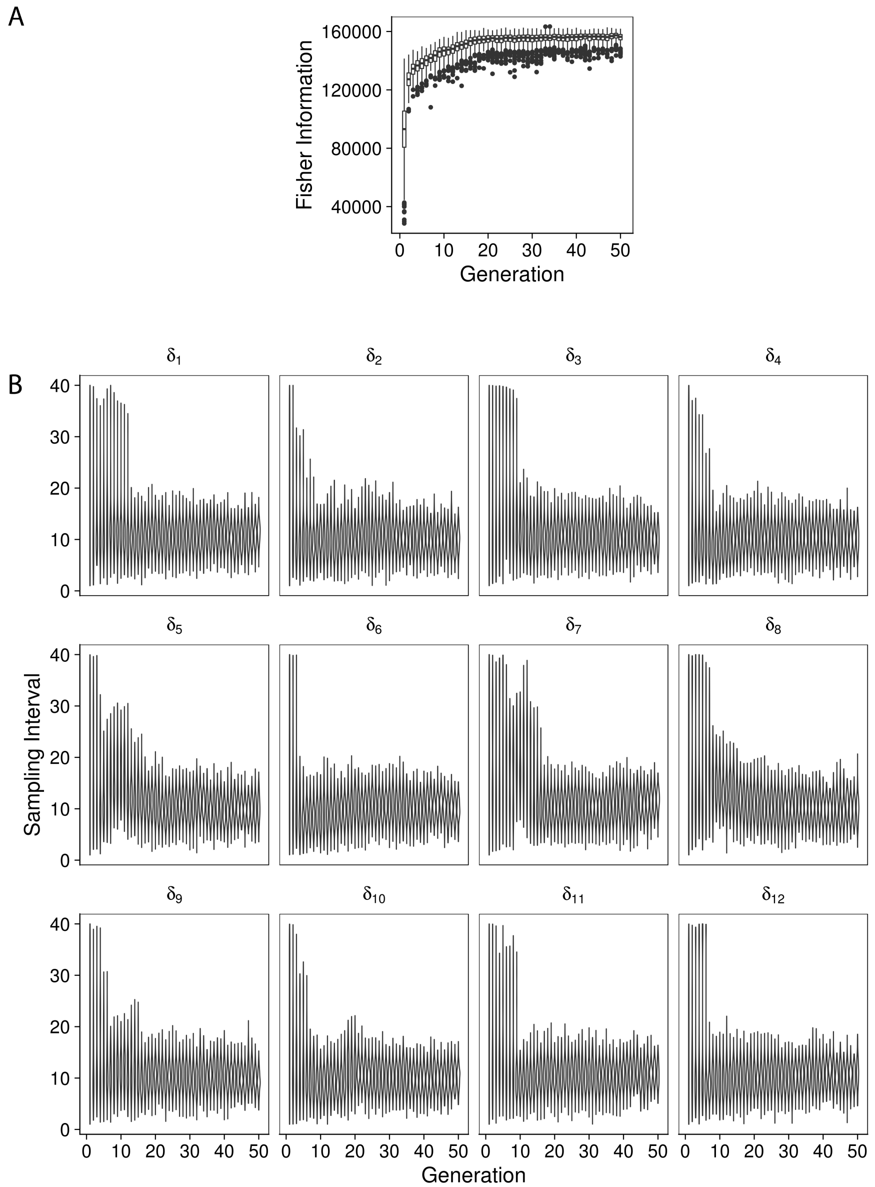

Fig 1. Accepted values of the sampling intervals for the PHN dataset with a variable sampling interval, as a function of the generation of the estimation algorithm. (A): The value of the Fisher information (summarised as a boxplot) for each generation, and (B) the parameter values accepted at each generation. The space appears well explored, and the solution appears converged after 50 generations.

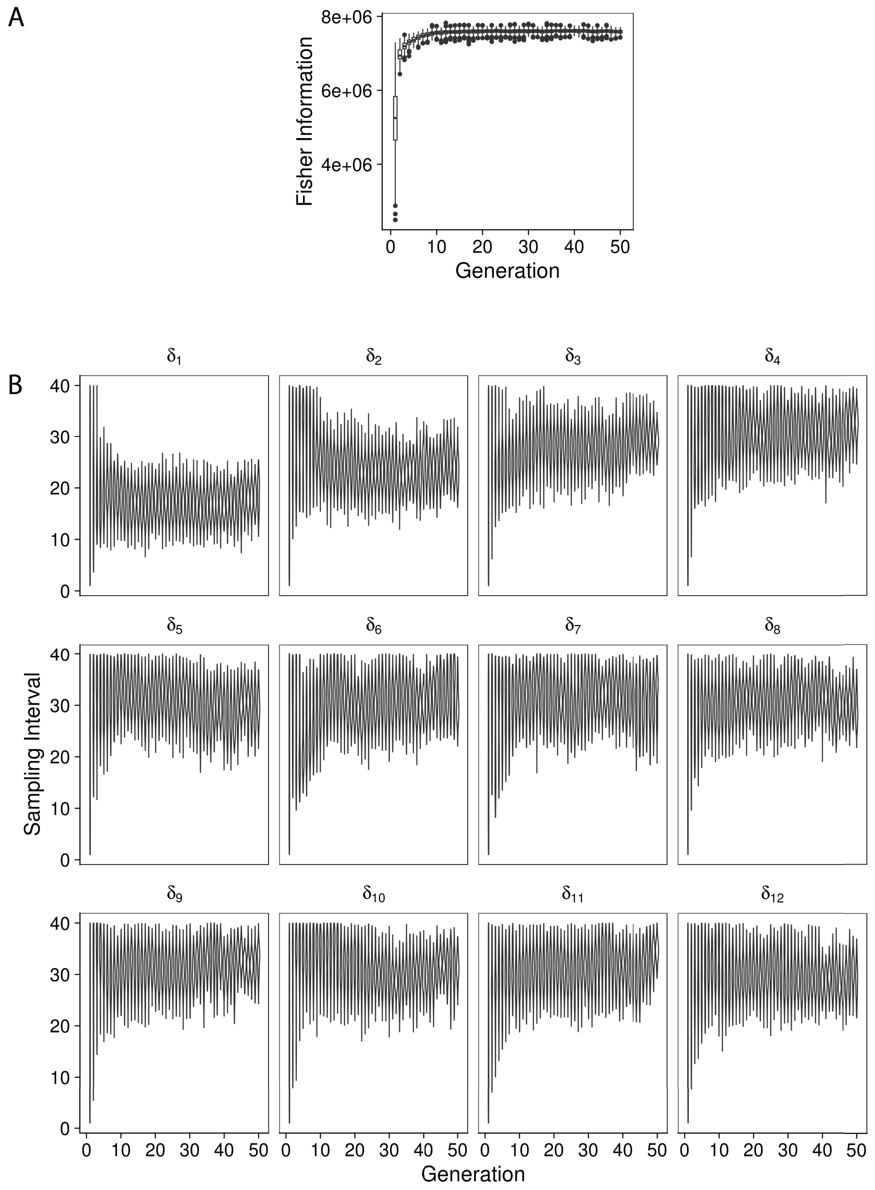

Fig 2. Accepted values of the sampling intervals for the HH dataset with a variable sampling interval, as a function of the generation of the estimation algorithm. (A): The value of the Fisher information (summarised as a boxplot) for each generation, and (B) the parameter values accepted at each generation. The space appears well explored, and the solution appears converged after 50 generations.

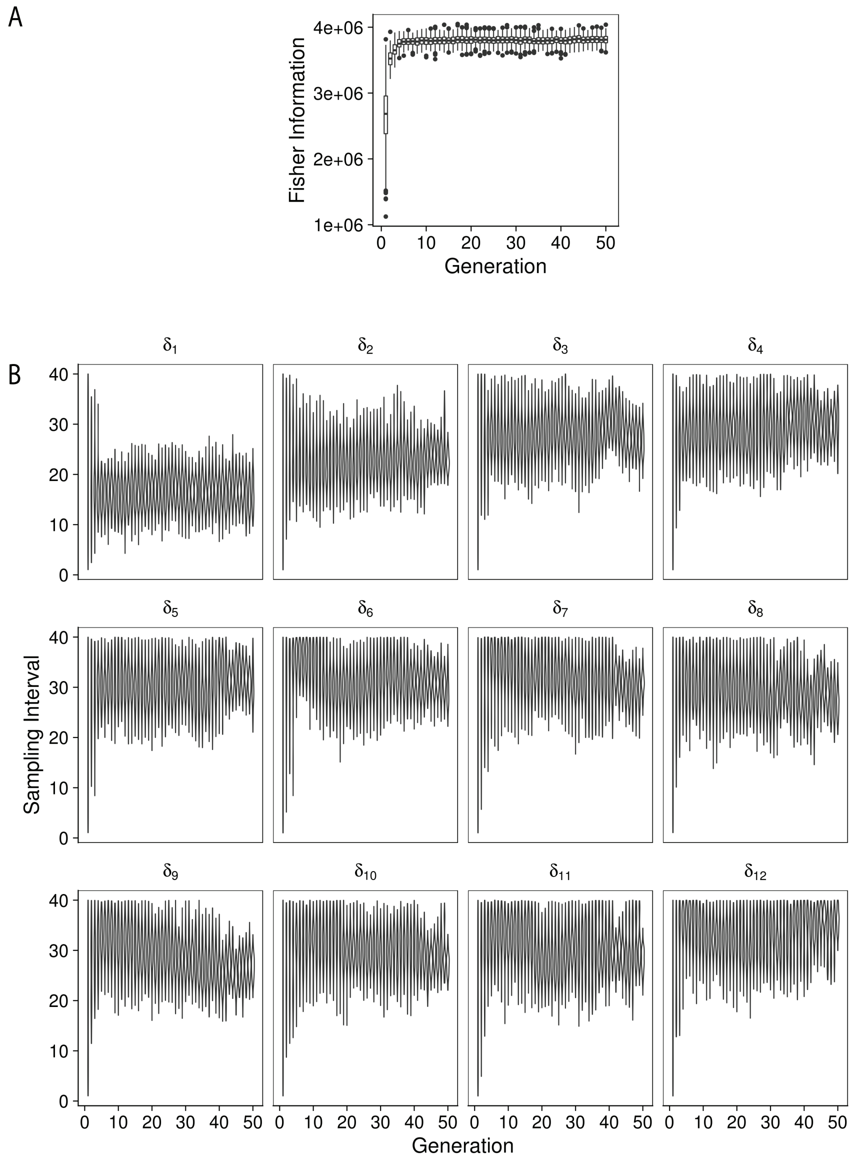

Fig 3. Accepted values of the sampling intervals for the combined dataset with a variable sampling interval, as a function of the generation of the estimation algorithm. (A): The value of the Fisher information (summarised as a boxplot) for each generation, and (B) the parameter values accepted at each generation. The space appears well explored, and the solution appears converged after 50 generations.

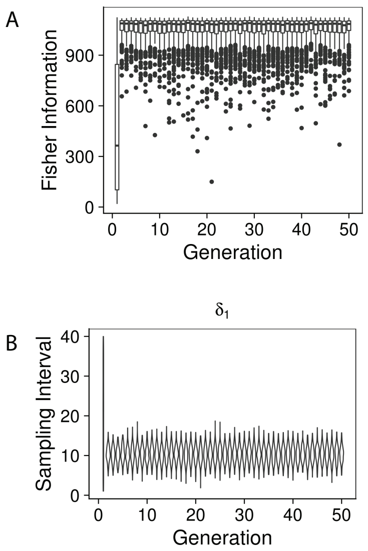

Fig 4. Accepted values of the sampling intervals for the PHN dataset with a fixed sampling interval, as a function of the generation of the estimation algorithm. (A): The value of the Fisher information (summarised as a boxplot) for each generation, and (B) the parameter values accepted at each generation. The space appears well explored, and the solution appears converged after 50 generations.

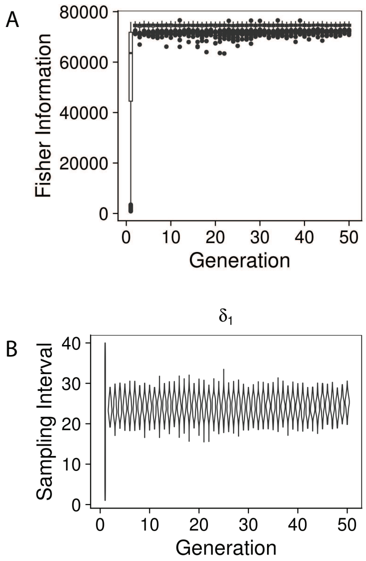

Fig 5. Accepted values of the sampling intervals for the HH dataset with a fixed sampling interval, as a function of the generation of the estimation algorithm. (A): The value of the Fisher information (summarised as a boxplot) for each generation, and (B) the parameter values accepted at each generation. The space appears well explored, and the solution appears converged after 50 generations.

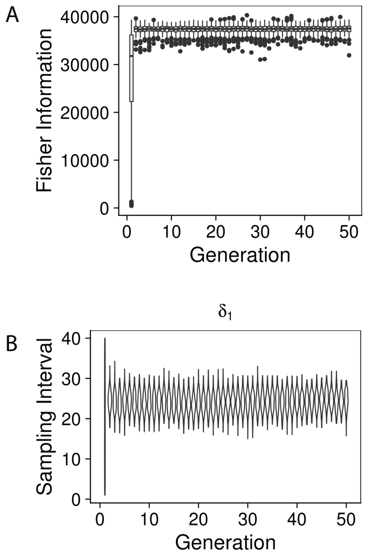

Fig 6. Accepted values of the sampling intervals for the combined dataset with a fixed sampling interval, as a function of the generation of the estimation algorithm. (A): The value of the Fisher information (summarised as a boxplot) for each generation, and (B) the parameter values accepted at each generation. The space appears well explored, and the solution appears converged after 50 generations.
