# Supplementary material for: Estimation of the force of infection and infectious period of skin sores in remote Australian communities using interval-censored data
Source: PLoS Comput Biol. 2020 Oct 5;16(10):e1007838. doi: 10.1371/journal.pcbi.1007838 (PMC7561265; doi:10.1371/journal.pcbi.1007838)
Supplement: S4 Text — Utility of the ‘conservative’ sampling strategy compared to the optimal sampling strategy. (PDF) [file pcbi.1007838.s004.pdf]

# Conservative sampling strategy

In Section Prospective Sampling Strategies, it was found that the optimal sampling strategy favoured a longer sampling interval in the absence of prevalence information. While this performed well for the relatively low prevalence observed in the setting where the HH dataset was collected, the strategy performed poorly in the higher prevalence setting where the PHN dataset was collected. A potential alternative strategy would be the *conservative* strategy, where the population is sampled every 10.45 days (as per the high prevalence setting). This strategy favours oversampling at the penalty of potentially broader credible intervals for parameter estimates in a low prevalence setting. A simulation estimation study of this, with 20 observations, is shown in Fig 1. The conservative strategy appears to perform well in the low-prevalence setting, although the variability in estimates is high (up to a 10% estimate error, compared with a 6% estimate error in the high prevalence setting). As this sampling strategy is optimal for the high prevalence setting, this study suggests that the conservative strategy may be a valid 'catch-all' strategy.

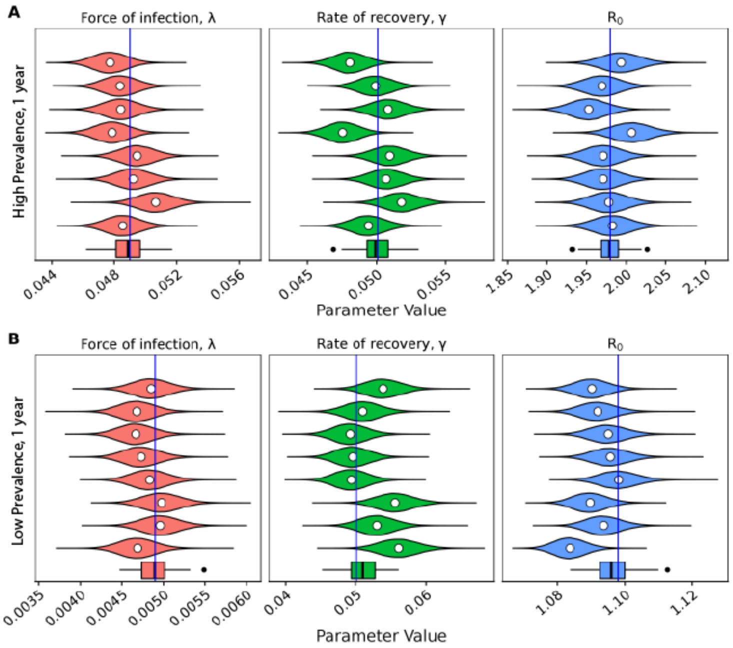


**Fig 1.** Marginal posterior distributions for the force of infection, $\lambda$, the rate of recovery $\gamma$, and the basic reproductive ratio, $R_0$, from 8 randomly generated populations from the linearised SIS model under the conservative sampling strategy (every 10.45 days, for 20 successive observations) under (A) a high prevalence setting, and (B) a low prevalence setting. The mean of each distribution is given by the white circle. The boxplot at the bottom of each panel represents the means of 64 marginal posteriors. The true value which was used to generate each population is represented by the blue line (high prevalence $\lambda = 0.049$, low prevalence $\lambda = 0.0049, \gamma = 1/19.97$)
