# Supplementary material for: Estimation of the force of infection and infectious period of skin sores in remote Australian communities using interval-censored data
Source: PLoS Comput Biol. 2020 Oct 5;16(10):e1007838. doi: 10.1371/journal.pcbi.1007838 (PMC7561265; doi:10.1371/journal.pcbi.1007838)
Supplement: S5 Text — Simulation study where the simulated data comes from a model with two infectious phases but is estimated using a single infectious phase. (PDF) [file pcbi.1007838.s005.pdf]

Model Sensitivity

To evaluate the method's performance on non-exponentially distributed sojourn times, we repeat the simulation experiment performed in Section Prospective Sampling Strategies but using a model with two infectious categories. The transitions of this model are shown in Table 1. Note that the mean infectious period is $2/2\gamma = 1/\gamma$, as is the case in the standard SIS model. The results of this simulation estimation and shown in Figure 1. In the daily observations case (panel A), the parameters are recovered successfully, although there may be a slight overestimation of the rate of recovery, γ. However, in the HH observations case (panel B), the force of infection, λ, and the rate of recovery, γ, are not successfully estimated. This is likely due to the smaller variance in the infectious period in the SII model compared to the standard SI model, which results in more frequent observations being required to see the transitions between individual states.

Table 1. Transitions of the SII model. The force of infection is given by λ, the transmissibility parameter by β and the rate of recovery by γ.

**Transition**	**Rate**
$(S, I1, I2) \to (-1, +1, 0)$	$\lambda := \beta(I1 + I2)$
$(S, I1, I2) \to (0, -1, +1)$	2γ
$(S, I1, I2) \to (+1, 0, -1)$	2γ

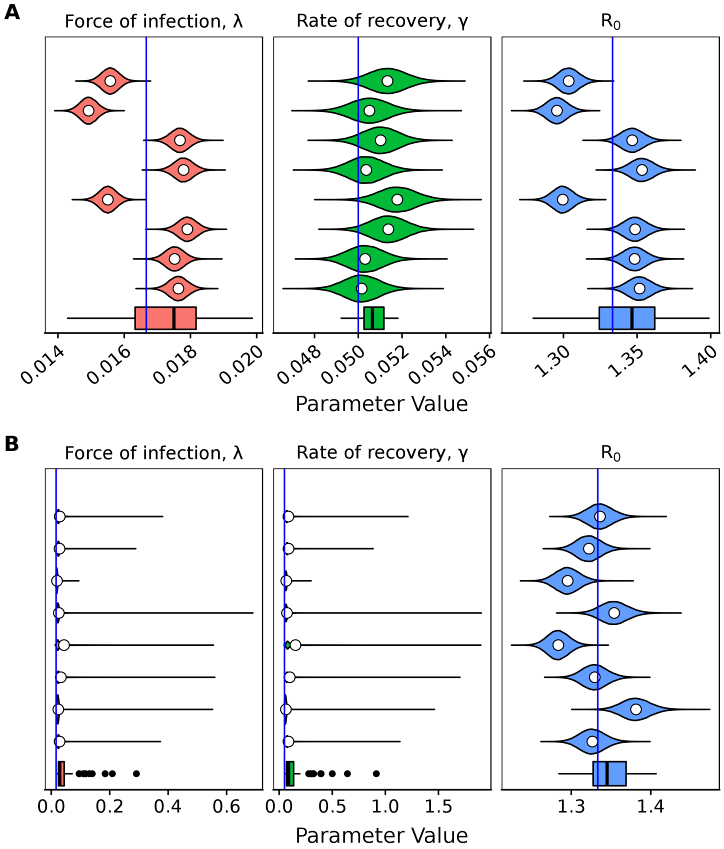

Fig 1. Marginal posterior distributions for the force of infection, λ, the rate of recovery, γ, and the basic reproductive ratio, R_0, from 8 randomly generated populations from the SII model under the empirical observation distribution using (A) daily observations and (B) according to the HH dataset, over 1 year. The mean of each distribution is given by the white circle. The boxplot at the bottom of each panel represents the means of 64 marginal psterior distributions. The true value which was used to generate each population is represented by the blue line ($\lambda = 1/60, \gamma = 1/20$). In the daily observation case, the parameters are able to be recovered successfully. In the observations according to the HH dataset, the force of infection, λ, and the rate of recovery, γ, are not precisely estimated.
