# Supplementary material for: Estimation of the force of infection and infectious period of skin sores in remote Australian communities using interval-censored data
Source: PLoS Comput Biol. 2020 Oct 5;16(10):e1007838. doi: 10.1371/journal.pcbi.1007838 (PMC7561265; doi:10.1371/journal.pcbi.1007838)
Supplement: Attachment — Submitted filename: parameterestimation-reviewed-responsetoreviewers.pdf [file pcbi.1007838.s009.pdf]

Reviewer #1

1.1: The analysis assumes an exponential distribution for the duration of sores. Is it possible to check this assumption? For instance, repeat the analysis with a more flexible distribution (Gamma/Weibull) and test whether the exponential distribution is supported by the data.

Without high resolution observations of a large number of individuals, it is not possible for us to verify the distribution for the duration of skin sores. To assess our method's sensitivity to distributional changes, we have added Appendix E in which we perform the simulation estimation experiment using an SII model (so the infectious period is Gamma distributed). The simulation estimation experiment does perform well with a high sampling frequency. Though, as one may expect, with the variance also requiring estimation if we expect a non-exponential distribution, we would need to sample a population more frequently (i.e., require more information).

1.2: The authors claim that is impossible to check whether a sampling scheme is ignorable? I do not see a reference, but I also wonder what they mean exactly with this. If having skin sores increases the rate at which individuals present to the doctor, one would expect that a statistical test whether the time between two subsequent visits is shorter in case of a negative visit followed by a positive visit compared to two negative visits. If there is a difference, this suggest that the sampling scheme is not ignorable.

Testing for whether the presence of skin sores changes the time to next presentation is not straightforward due to missing data. If, on every presentation by an individual, their skin sore status was noted, then we could have faith in the empirical time between observations as some measure of whether the sampling scheme is ignorable. However, skin sores are common, and relatively minor, and so the status is not always noted in the clinical record. As an example, there are 432 records where a positive entry is followed by a 'missing' entry in less than 2 days' time.

If we ignore the missing entries and test the empirical difference (accounting for individual effect), then there is a significant difference ($p = 0.028$, Student's t-test) between presentation times, with negative followed by positive being longer (mean of 27.2 days) compared with negative followed by negative (mean of 23.2 days).

We have added a reference to work that demonstrates that it cannot be proven whether a sampling scheme is ignorable. We have added the following to the text to help clarify the point around measuring presentation times (Line 169):

"If we ignore these `missing' entries, the mean time to next positive presentation following a negative presentation is 27.2 days, while the mean time to next negative presentation following a negative presentation is 23.2 days. If skin sores caused more frequent doctors presentations, then we would expect these numbers to be reversed. However, the empirical mean time to next presentation is sensitive to the frequency of missing data, and so it is unclear whether this difference can be attributed to a change in patient behaviour based on infection status, or limitations in data collection" (Second to last paragraph, Data Structure).

1.3: In the description of the data structure, I had the impression that not all information was used. The probability P(X_{i,1}=0)=\gamma/(\lambda+\gamma) and P(X_{i,1}=1)=\lambda/(\lambda+\gamma). Later on, the authors discuss that the recovery rate for participants may be higher than for the general population due to treatment, but I think an earlier reference is useful.

We are unclear as to what information the reviewer suggests is not being used – the only information available to us is whether or not an individual is susceptible or infected with skin sores.
We have added the following sentence to the "Data Structure" section (Line 175):
"It is important to note that in the settings studied here, treatment is routinely offered and applied to skin sores. This will augment the estimate of the recovery rate, γ, to be the average duration of infection in the presence of treatment. However, as treatment is routinely applied in many settings where skin sores are endemic, this estimate is still relevant when considering control schemes and survey designs."

1.4: Related to the previous point, I do not really understand how the results in Table 2 relate to the formulas on page 3: The model estimates \lambda and \gamma. Is R0 determined based on the prevalence at first presentation (as the recovery rate may be higher for individuals in the study due to treatment) or based on the estimates of lambda and gamma alone. If so, why are the data of the first presentation not used to obtain a better estimate of lambda and gamma
R0 is determined using Equation (3). Since lambda and gamma are correlated, we use the formula (Eq 3) for each sample from the posterior distribution to evaluate the posterior distribution for R0. It would be invalid to compute R0 from the reported marginals for each of lambda and gamma as this would ignore the correlations.
We have added the following sentence to the end of "Data Structure" (Line 184):
"To calculate the basic reproductive number, R_0, we apply the formula in Eq 3 for each sample from the posterior distribution."

1.5: When I read that the authors linearise the SIS-model about the endemic equilibrium, I expected a different analysis than the very basic two-state Markov model they are using in which there is no dependence between individuals.
The linearization does indeed result in a simple mathematical expression. We have explored its validity and application in this article.

1.6: Page 6, I think the authors should mention the priors they use when they mention the MCMC procedure.
Thank you for highlighting this oversight – we have added the following sentence to the end of the "Data Structure" section (Line 182):
"We use truncated normal distributions, TN(0.5, 0.5), truncated at 0, as priors for both the force of infection, \lambda, and the infectious period, \gamma."

1.7: On Page 7, verification of linearization procedure? Did the observation start immediately after the seeding? Was there a conditioning on non-extinction?
The model was run for 10 years before observation started. There was no explicit conditioning on non-extinction, although it should be noted that in none of the samples did stochastic extinction occur, likely due to the high initial prevalence. We have added the following sentence to the first paragraph of the "Verification of linearisation procedure" section (Line 231):
"The system is run for 10 years before observation begins." and "No stochastic extinction occurred in any of the simulations throughout this work."

1.8: More importantly, I do not really see the need for the linearization? The matrix exponent of a sparse 301x301 matrix takes 0.2 seconds on my laptop. This would mean that 40,000 iterations take a bit more than 2 hours, which is not really prohibitive.

In the standard (non-linearised) SIS model with 301 individuals, we require the prevalence, *I,* at every time, *t.* Thus, the size of the state space (and hence dimensions of the matrix to be exponentiated) is 301^2 = 90601. The matrix is sparse, but exponentiation gives a "Problem too large" error in R, and I was not able to successfully exponentiate in MATLAB.

1.9: I also do not understand the logic behind the need to verify the presentation distributions. Would a direct analysis, without any checking, not already tell you whether there is sufficient information in the data? (Based on credibility intervals for instance).
While tight credible intervals and good convergence criteria suggest that the estimation algorithm is working well, this does not account for the model stochasticity. That is, one could obtain a wide credible interval for a particular model-data combination, as there is a lot of variability in the system. Performing this check allows us to assess what level of uncertainty we might expect for these different presentation distributions when we are certain that the data are truly generated from this model, under each of these sampling mechanisms. We also find it useful to demonstrate that, for a wide observation interval (such as in the RC dataset), the algorithm reports a wide credible interval, rather than a tight but incorrectly centred credible interval.

1.10: Regarding the recommended sampling strategies, is there a rule of thumb, for instance, the time between samples should be in the order of 1/\gamma, such that the simulations do not have to be performed for each value of \lambda and \gamma?
To our knowledge, no such rule of thumb currently exists. Thinking more generally about sampling dynamic processes, sampling should ideally occur at least twice as fast as the fastest process (the Nyquist rate). However, as lambda is proportional to the prevalence, it may not be possible *a priori* to know which process is the fastest, and this may change as prevalence changes. For nonlinear models, the optimal sampling strategies are a function of the parameter values, and without performing an extremely comprehensive investigation of the designs resulting from different parameter combinations, it could be misleading to make any sort of recommendation. Verifying any such generalised relationship between the sampling interval and 1/\gamma is beyond the scope of this work.
We make the code available for researchers to evaluate their own strategies, given their own understanding of the parameter values (i.e., prior distributions) in order to make the best decisions in each case.

Reviewer #2

2.1: This is an interesting and helpful analysis of a difficult problem in infectious disease epidemiology, namely how to estimate transmission rates for endemic infections from interval-censored panel data. My primary concern with this analysis relates to the fact that asymptomatic colonization is typically assumed to be a precursor to symptomatic disease, and the SIS framework employed does not allow for colonization to impact transmission. Given that all available evidence suggests that asymptomatic individuals transmit at a rate similar to those with invasive infection. Given that this is the case, how should the estimates of R0 provided by the authors be interpreted? In particular, given that asymptomatically colonized individuals may be infectious for long periods of time, this may result in a downward bias in the estimate of the infectious period and an upward bias in the estimated force of infection at each time point. The authors should address whether this omission of colonization impacts their R0 estimates in order to ensure that their results are reliable and clinically useful.

With that said, I believe this issue is addressable via some additional assumptions about the relationship between the equilibrium prevalence of invasive disease and the equilibrium distribution of prevalence as well as the duration of colonization in the absence of invasive disease.

Other than these concerns, I do not have any other major questions about their analysis and found the section on optimal sampling to be a useful complement to the transmission modeling.

Skin sore pathogens are known to colonise the upper respiratory tract and skin, and individuals in these states may also contribute to transmission. The relative contribution of these states to secondary infection spread is a source of active enquiry in related work being undertaken by our group but is presently poorly understood. Given the hyperendemic prevalence of skin sores in this setting, and the high observed infectiousness of skin-to-skin transmission, as well as the absence of definitive evidence on the role of strains, reinfection and colonization in driving transmission and immunity, we take the simple approach of modelling transmission of skin sores in the absence of a carrier state.

We note the recent work of Chisholm et. al (*Implications of asymptomatic carriers for infectious disease transmission and control*, Open Science 2018) which shows that ignorance of carriage can lead to interventions causing an *increase* in prevalence under certain circumstances. We noted previously the role of carriage in the discussion (Line 365), and have updated the final sentence of this paragraph:

"Given the hyperendemic prevalence of skin sores in this setting, the observed high infectiousness of skin to skin transmission, and in the absence of longitudinal data related to carriage available in this study, we have ignored the carrier state in this model. However, we note that extension in this area represents an important path for future work."

2.2: P(t) is not defined as a probability anywhere; including this information would be helpful just from a clarity perspective.

The matrix P(t) is not a probability itself, but rather, when multiplied by a probability vector (e.g. the initial state), becomes a probability vector.

Reviewer #3

3.1: The observed outcome are skin sores (present/absent at the time of examination). Yet, as stated by the authors at the beginning of their introduction, both S. aureus and S. pyogenes (GAS) can cause these lesions. Within the GAS species, multiple (unrelated) serotypes are likely circulating, and there is no molecular information available to assume that one single strain is being transmitted in the communities under study. Similarly, data relative to asymptomatic colonization are absent, so the authors should be extremely careful when discussing insights into GAS transmission.
I think reformulating the last sentence of the abstract as the last sentence of the Conclusion (leaving out GAS) and clarifying this point further with a paragraph in the Conclusion (around line 343, where the immunity and carriage are discussed) is necessary. Typically, the whole picture is likely much more complex, with multiple infections by the same serotype conferring immunity to that serotype (Pandey et al. Streptococcal Immunity Is Constrained by a Lack of Immunological Memory following a Single Episode of Pyoderma, 2016, PLoS Patog.), but not at all to other strains circulating. I do not advise to advance such speculations and do not see how they could improve their model in that direction with the data available, but they should clarify the limitations of having a symptom of infection (skin sores) as outcome, rather than the actual pathogen identification.
Multiple studies in this setting have identified Group A Streptococcus as the dominant skin pathogen in this context, often accompanied by secondary Staphylococcal infection. Given the consistency of observed relationships between the two, we consider 'skin sores' as the syndromic condition transmitted from one individual to another.

We have added the following paragraph to the conclusion (line 380):
"It must also be noted that the condition of skin sores can be caused by a number of pathogens. The microbiology of skin sores in the Northern Territory, Australia, has been studied previously [40], and found that *Streptococcus Pyogenes* remained the dominant pathogen, but co-infection with *Staphylococcus aureus* was present. Without microbiologic information present in our data sets, we are unable to determine which pathogen is causing infection. Rather, we see these results as a quantification of skin sores as a general condition."

We note the reviewer's comments on immunity, particularly in the context of a highly strain-diverse setting and agree that speculations around the role of immunity are outside the scope of this work.

3.2: Unfortunately, as stated by the authors in their conclusion, the condition of disease dynamics equilibrium on which the analysis is based is likely to be violated in real-world settings. This weakens the plausibility of the estimations obtained.
Is there any evidence in one of the three setting under observation that this condition was likely fulfilled during the period of the data collection?
Data on prevalence of skin sores and scabies is not collected for periods outside of the given studies, so we cannot say for certain the impact of the program relative to a known baseline. While for all endemic infectious diseases variability is observed over medium to long time cycles, the hyperendemic prevalence of skin sores in the remote communities under study has remained stubbornly consistent for decades despite intervention (Andrews et. al *A Regional Initiative to Reduce Skin Infections amongst Aboriginal Children Living in Remote Communities of the Northern Territory, Australia*, PLoS Negl Trop Dis 2009).

3.3: Are the patient ages at presentation known?
If yes, why an age structure was not considered, given that impetigo is age-dependent?

Patient ages are available in the datasets. Age structure was not considered for two reasons:
1) Inference on these datasets is already challenging in the absence of age structure, and this work represents the first time these parameters have been estimated at all, and
2) In the context of control, it is really the high prevalence age groups that are most important for designing surveys and intervention measures. We explicitly note the absence of age structure in the first paragraph of Section 3, and the paragraph in the conclusion starting on line 395.

3.4: In the Supporting Information section, the file "Patient ages at presentation" does not correspond to their actual age but empirical observation times. This should be corrected to avoid confusion. In the conclusion the higher prevalence/force of infection in the young children dataset could be further discussed.
Thank you for highlighting this discrepancy, we have corrected the column name to be "time_difference" to accurately reflect the column being the time between successive visits rather than empirical age.

3.5: The choice of the parameter's values to simulate the population requires argumentation. A table summarizing these parameters and justifying the chosen values (with citations if necessary) would be helpful for the reader.
There is only one set of parameters that is used in the simulation estimation section: lambda = 1/60 and gamma = 1/20. These parameters were chosen in an informed but arbitrary manner, and no citation can be given. We did also test parameter sets resulting in 'high' and 'low' prevalence (Appendix D) but again these parameters were not chosen to accurately represent any real dataset.

We have added the following sentences to the Analysis section (Section 4) (Line 190):
"To explore the suitability of the methods and available datasets, we choose reasonable values for the force of infection, lambda, and the rate of recovery, gamma. In the main text, we use lambda = 1/60 and gamma = 1/20, while Appendix D presents results for different values. After these verifications, the estimation method is applied to the observational data."

3.6: Line 22, I suggest using "prior generation" instead of "generation prior" for clarity sake.
Thank you – we agree and have edited the text.

3.7: Line 157 "It is noted that the estimate of infectious period in any modern setting will be augmented by treatment". This sentence is unclear, no reference is given. Intuitively I would agree with the opposite: "Thus, this estimate of the infectious period is influenced by treatment, and so is likely to be lower than the natural infectious period" (Line 351). The authors should clarify.
We have updated this sentence to read (Line 157) "Further, it is noted that the estimate of infectious period in any modern setting will be reduced in the presence of treatment."

3.8: Line 161 "It is important to note that it has been proven impossible to test whether or not a sampling scheme is ignorable." This is a very strong statement, again lacking a reference to support it. The authors should reformulate this sentence and provide a reference.
We have addressed this comment as per Reviewer #1's comment (1.2).

3.9: Line 219 "The results are visually similar". This statement could be more quantitative.
We have updated this sentence:

“The procedure re-estimates parameters well and in an unbiased manner, with the true parameters being within the 95% confidence interval, similar to that seen in Fig 2 under the same observation distribution.”

3.10: Line 230 “However, as the prevalence is observed at each survey visit,”. This sentence is unclear to me.
We have updated this sentence to the following:
“However, at each survey visit, the data contains the number of individuals currently infected, and thus an estimate of the prevalence. As the basic reproductive ratio, R_0, is directly related to the prevalence, it is still possible to estimate the basic reproductive ratio, R_0, in this setting.”

3.11: Figure 1. Visualizing the population age distributions for each dataset on the side would be helpful
We appreciate this suggestion, and have added this as a second panel to the figure.

3.12: Figure 2. Adding a y-axis title, such as Daily observations, 1 year/PHN empirical observation distribution, 1 year to differentiate plot A from plot B at first glance would be helpful.
We have added subtext labels to each of the violin plots contained in the paper to help with clarity.

3.13: Visualizing the observation distribution from the RC dataset would already highlight the point made in the following figure (even if they are far from the true values, as expected). As such, its inclusion in the figure would be welcomed.
The simulated values for the RC dataset are so far away from the true values that it requires either a) a different x-axis, which appears misleading, or b) means that any detail in panel A and panel B is lost as it is squashed to the left-hand side of the figure. After some consideration, we have chosen not to update the figure. We have added the following to the figure caption for clarity:
“The observation distribution from the RC dataset was tested, but has not been visualised as the estimates were far from the true values (See Fig 3).}”

3.14: Figure 3: The unit [days] on the x-axis are missing. Furthermore, visualizing the corresponding time to horizon (although the calculation is as simple as Sampling interval x 20) would be helpful for the reader.
We have updated the units. As the time to horizon is “sampling_interval * 20” as noted by the reviewer, we have added this note to the Figure 3 caption:
“Each realisation contains 20 observations from the simulated population, leading to the time horizon for each realisation being 20 x sampling interval.”

3.15: Figure 4: Using the same x-axis values as in Figure 2 would ease comparison of the two figures.
We have updated Figure 4 to have as similar tick values as possible to that of Figure 2.

3.16: The legend of Figure 5 is identical to that of Figure 2, except that in legend 2, Marginal posterior distributions are mentioned while marginal posteriors are mentioned in legend 5. From my understanding, Fig 2B and Figure 5A are redundant (just posterior distributions obtained from different randomly obtained populations) is that correct? Or is there any conceptual difference between these? If yes, it is unclear. Again, labeling the y-axis would enhance the clarity of the figure at first glance.

Figure 2B and 5A are posterior distributions obtained from different randomly obtained populations. They are presented twice (and in different orders) to highlight differences between i) daily observations and more spaced out observations (pre-empting the optimal design section) and ii) certainty of estimates in two of the three considered datasets. To clarify these figures, we have added subtext to the A and B labels to all violin plots which show the source dataset.

3.17: Figure 6. Visualizing the same plot for the HH dataset (even though it might look like the PHN one) would add some support for the reader.
We have added the extra panel to this figure.

We thank the reviewers once again for their time and insightful comments that we believe have helped to improve the manuscript quality.
