# Supplementary material for: Estimation of the force of infection and infectious period of skin sores in remote Australian communities using interval-censored data
Source: PLoS Comput Biol. 2020 Oct 5;16(10):e1007838. doi: 10.1371/journal.pcbi.1007838 (PMC7561265; doi:10.1371/journal.pcbi.1007838)
Supplement: Attachment — Submitted filename: ParameterEstimation-ResponseToReviewers2-200221.docx [file pcbi.1007838.s010.docx]

Dr Michael J. Lydeamore

Department of Infectious Diseases

The Alfred & Central Clinical School

Monash University

[michael.lydeamore@monash.edu](mailto:michael.lydeamore@monash.edu)

3^rd^ March 2020

Dear Editor,

We sincerely thank you and the three reviewers for the time and insightful comments provided on our manuscript titled ‘Estimation of the force of infection and infectious period of skin sores in remote Australian Aboriginal communities’.

We have addressed the comments made by the reviewer with remaining queries. In particular, we have clarified the language around the impact of treatment on our estimations, and our view on the question of the distribution of the infectious period.

Please find on the following pages a detailed response to the referee comments. We hope that with these revisions you will find our work suitable for publication in PLoS Computational Biology, and we look forward to hearing from you.

Kind regards,

Michael J. Lydeamore

(On behalf of Patricia Campbell, David Price, Yue Wu, Adrian Marcato, Will Cuningham, Jonathan Carapetis, Ross Andrews, Malcolm McDonald, Jodie McVernon, Steven Tong and James McCaw)

**Reviewer #1**

1.1: To obtain a reliable non-parametric estimate of the distribution of the duration of the infectious period, may require more data than is available. However, one could use another family of distributions and test which distribution gives the best fit (using e.g., AIC/DIC). This at least gives some hints whether the assumption of the exponentially distributed infectious period makes sense.

Bowen et al. (2014) performed a trial to assess the time to clear skin sore infection under different antibiotic treatment regimes. The treatments were assessed non-inferior, and having combined the results, treatment success was identified in 40-53% of patients by day 2, and in approximately 85% by day 7. Isolation of Streptococcus pyogenes reduced from around 85-95% on Day 0 to around 50% on Day 2, and less than 10% by Day 7.

In our study, we do not have data on those that were and were not receiving treatment, but it is expected that a portion of the population were prescribed and used antibiotics to treat their skin sores, which would result in a time to clear infection consistent with those in the Bowen et al. study above (e.g., an exponential distribution with rate parameter ~0.3). The remainder of the population are likely to have not received treatment, resulting in the extended duration of infectiousness that we have observed. As our data are a combination of treated (with short clearance time) and untreated (with a longer clearance time), we propose that an exponential distribution for the duration of infectiousness is a parsimonious choice. Further, we agree with the reviewer that we are unlikely to have a sufficiently fine observation schedule to be able to establish differences between any proposed distributions.

Without solid justification for an alternate distribution – of which there are many choices – it seems unfair to arbitrarily choose one or two further distributions to test. To specify a single model with one of these alternate distributions for the duration of infectiousness, fit it to these data in a Bayesian framework, so as to evaluate the model fit, is no small task. In fact, the likelihood for these models is complex (hence the linearised approach adopted in this paper), and would result in significant additional computational/modelling effort – greater than that which has gone into the manuscript thus far. We have presented a section on proposing future sampling strategies to best estimate the model parameters, and a similar approach could be taken to best discriminate between different models should one wish to establish the most appropriate model structure as a future study (e.g. Hainy et al. 2019).

We have added to the manuscript the following text in the Discussion (Line 352):

“Previous work on the duration of skin sore infection has estimated that under treatment, skin sore infections clear in approximately 50% of individuals in 2 days, and 85% of individuals in 7 days [37]. In this study, we do not have information on whether or not an individual was prescribed treatment, but it is expected that a proportion of the population were prescribed and used antibiotics. For the remainder of the population who were not treated with antibiotics, it is expected that their clearance time would be longer. As our data are a combination of treated and untreated individuals, we propose that an exponential distribution for the duration of infectiousness is a parsimonious choice. Further, without more frequent observations, we are unlikely to be able to distinguish between other proposed distributions with confidence (Appendix E). Should accurately characterising the distribution of the duration of infectiousness be of interest, a similar approach to that in Section 4 could be used to design a study to best discriminate between different models [38]”

1.2: In [27], it is shown that based on data it can never be proven that a scheme is ignorable, i.e., there always exists a stochastic process for which the sampling scheme is ignorable. However, in this setting, the stochastic process is given (up to the values of the parameters, i.e., it is an SIS-model). Given the stochastic process, one can test whether the scheme is ignorable, so I think the stressing of the fact that one cannot determine whether a sampling scheme is ignorable is misleading in this case.
I do like the addition on the between-presentation times.

We have removed the sentence “It is important to note that it has been proven *impossible* to test whether or not a sampling scheme is ignorable”.

1.3: I meant that if the status at the first presentation is known, this contains also information.
If the system is in equilibrium (as is assumed), the probability that a patient is positive is \lambda/(\lambda+\gamma). This information is not used in the likelihood. Later on, the authors argue that the recovery rate for participants may be higher than for the general population due to treatment, and hence, the \gamma may change once the participant enters the cohort, but this is not discussed when the likelihood is created.

We have introduced an individual-level model of the transmission dynamics. In a population level model, one does need to include a term to represent the initial conditions of the system (i.e., corresponding to the number of infectious individuals at time 0). Here, at the individual level, this would correspond to whether the individual is infectious at the beginning of the study, as the reviewer has noted is true with probability \lambda/{\lambda+\gamma). Our two state system considers transitions from S -> I or I -> S, and so the transition from the 1^st^ observation time to the 2^nd^ observation time (from X_{i, 1} to X_{i, 2}) accounts for their status at time 0. That is, for individual i, P_{X_{i, 1}, X_{i, 2}}(t_2-t_1) would be dictated by whether individual i was infectious or susceptible at time 0 (i.e., their initial visit), which is selected from the relevant entry in Eq (1). Given a sufficiently large population size, these terms will contribute to a numerical contribution to the likelihood as per the formula noted by the reviewer.

Regarding the recovery rate, as discussed in our response to query 1.1 not all individuals in the cohort received antibiotics, and of those that did, we have no way to identify what the antibiotic was or how it was used. Further, we note that the interpretation for \gamma is different to a traditional ‘natural history’ infectious period, due to the partial presence of treatment and the endemicity of disease in this setting. We have clarified this on line 386 and added the following sentence:

“In the populations in which these data were collected, treatment is routinely administered for skin sores. Thus, these estimates of the infectious period are inclusive of the uptake and effect of treatment, and so are likely to be lower than the *natural* infectious period (that is, in the absence of treatment being available). Although this interpretation of the infectious period is different to the *natural* infectious period, it is arguably more useful in an epidemiological context, as treatment will be prescribed in any modern setting for a skin sores infection, and particularly for settings in which skin sores are highly endemic.”

1.4: In line 52, the authors mention that the dimension of the state space is N, I think it should be N+1 (there can be 0, 1, …., N infectious individuals).
When I commented on the fact that taking the matrix exponent of an NxN-matrix is very doable when N=300, the authors replied that the dimension is actually 301^2x301^2. Why is this not corrected in the text? I also do not understand why the dimension should be 301^2x301^2. If each individual is explicitly present, I would expect 2^{N} different states and not 301^2.

We apologise for the oversight in the dimension statement in the text, and our error in the combinatorics for the size in the state space. We have updated the text as follows:

“For the SIS model with individuals explicitly stated, the size of the required state space is $2^N$ \cite{Keeling2008}. When constructing the Markov chain representation of the SIS model then, the generator matrix, $Q$, is $2^N \times 2^N$, meaning that for large numbers of individuals, computing the matrix exponential $\exp(Qt)$, is computationally intractable.”

1.5: I noticed that the notation used in section 2 is confusing. In Table 1, S and I represent the number of individuals who are susceptible and infectious, respectively. However, the quasi-equilibrium I^* assumes that I is the fraction of the population, also the formula for R_0 assumes that the force of infection is \beta I with I the fraction of the population who is infectious, (if I is the number of infected individuals, R0=\beta N/\gamma). The authors should stick to a single interpretation of I and S (either numbers or fractions) and use a different symbol (e.g., i and s) for the other.

We thank the reviewer for this observation. We have updated the notation in Section 2.1, using i = I/N, and updated the infection transmission in Table 1 to be \lambda = \beta I / N.

1.6: The numbering of the sections is strange, there are two sections 2.1 for instance.

We have double checked the section numberings and haven’t found any duplicate sections. We suspect this may have been due to some formatting issues in a tracked-changes version of the manuscript. We apologies for the inconvenience.

1.7: “The first of these conditions effectively means that the probability that an individual is either susceptible or infectious at time tj , given all past information, is independent of tj ,and all past examinations.” To me, this is not what the first condition means. It says that the infection status at time t_{i,j} only depends on the status at time $t_{i,j-1}$ and on $t_{i,j-1}$ and $t_{i,j}$, but not on whether is a sampling at time $t_{i,j}$ or on previous sampling times. It is, however, dependent on the time since the last known status, so it does depend on t_j.

We thank the reviewer for this suggestion and have updated this sentence as follows:

“The first of these conditions means that the infection status at time $t_{i,j}$ only depends on the status at time $t_{i,j-1}$ and on the duration $t_{i,j-1}$ to $t_{i,j}$, but not on whether the individual's infection status is sampled at time $t_{i,j}$ or on previous sampling times.”

We thank the reviewers once again for their time and insightful comments that we believe have helped to improve the manuscript quality.
